# Plasma Amino Acid Abnormalities in Chronic Heart Failure. Mechanisms, Potential Risks and Targets in Human Myocardium Metabolism

**DOI:** 10.3390/nu9111251

**Published:** 2017-11-15

**Authors:** Roberto Aquilani, Maria Teresa La Rovere, Daniela Corbellini, Evasio Pasini, Manuela Verri, Annalisa Barbieri, Anna Maria Condino, Federica Boschi

**Affiliations:** 1 Dipartimento di Biologia e Biotecnologie Università degli Studi di Pavia, Via Ferrata, 1, I-27100 Pavia, Italy; dottore.aquilani@gmail.com (R.A.); manuela.verri@unipv.it (M.V.); 2 Istituti Clinici Scientifici “Maugeri”, IRCCS, Divisione di Cardiologia, Centro Medico di Montescano, Via per Montescano, Montescano, 35, I-27040 Pavia, Italy; mariateresa.larovere@icsmaugeri.it (M.T.L.R.); daniela.corbellini@icsmaugeri.it (D.C.); 3 Istituti Clinici Scientifici “Maugeri”, IRCCS, Divisione di Riabilitazione Cardiologica, Centro Medico di Lumezzane, Via G. Mazzini, Lumezzane, 129, I-25065 Brescia, Italy; evpasini@gmail.com; 4 Dipartimento di Scienze del Farmaco, Università degli Studi di Pavia, Viale Taramelli, 14, I-27100 Pavia, Italy; annalisa.barbieri@unipv.it (A.B.); annamaria.condino@unipv.it (A.M.C.)

**Keywords:** CHF, NYHA classes, left ventricular function, arterial amino acids, nutritional adequacy

## Abstract

The goal of this study was to measure arterial amino acid levels in patients with chronic heart failure (CHF), and relate them to left ventricular function and disease severity. Amino acids (AAs) play a crucial role for heart protein-energy metabolism. In heart failure, arterial AAs, which are the major determinant of AA uptake by the myocardium, are rarely measured. Forty-one subjects with clinically stable CHF (New York Heart Association (NYHA) class II to IV) were analyzed. After overnight fasting, blood samples from the radial artery were taken to measure AA concentrations. Calorie (Kcal_I_), protein-, fat-, carbohydrate-intake, resting energy expenditure (REE), total daily energy expenditure (REE × 1.3), and cardiac right catheterization variables were all measured. Eight matched controls were compared for all measurements, with the exception of cardiac catheterization. Compared with controls, CHF patients had reduced arterial AA levels, of which both their number and reduced rates are related to Heart Failure (HF) severity. Arterial aspartic acid correlated with stroke volume index (*r* = 0.6263; *p* < 0.0001) and cardiac index (*r* = 0.4243; *p* = 0.0028). The value of arterial aspartic acid (µmol/L) multiplied by the cardiac index was associated with left ventricular ejection fraction (*r* = 0.3765; *p* = 0.0076). All NYHA groups had adequate protein intake (≥1.1 g/kg/day) and inadequate calorie intake (Kcal_I_ < REE × 1.3) was found only in class IV patients. This study showed that CHF patients had reduced arterial AA levels directly related to clinical disease severity and left ventricular dysfunction.

## 1. Introduction

The human heart uses large amounts of amino acids (AAs) as regulators of both myocardium protein turnover [1,2,3] and energy metabolism [4,5,6,7,8], but uses few AAs as substrates for direct energy production [6]. The heart’s reliance on AAs increases during heart failure (HF) because of high myocardium anabolic activity and cardiomyocyte energy shortage [9]. Anabolic activity of the ventricle wall is induced by both high levels of ventricular pressure [3,10] and a myocardial substrate shift from fatty acid oxidation (FAOX) to glucose oxidation (GLUOX) [11,12].

In healthy hearts, intermediaries of tricarboxylic acid (TCA) cycle for energy production consist exclusively of AAs [5]. Even in early HF, in which energy deficiency occurs, there are fewer intermediary metabolites, whereas cardiomyocyte content in AA aspartic acid increases [13]. These AA serve physiologically to stimulate mitochondrial energy production under anaerobic conditions as well as contributing to replenishing the TCA cycle [6,7,8], thus assuming an important pro-energy role.

Crucial for heart AA availability, the take-up of AAs by the myocardium depends solely on arterial AA levels [1,14,15]. Myocardium branched chain AA (BCAA) content, the strongest heart anabolic agent [3,16], is closely linked with arterial BCAA levels and does not depend on insulin [14] in any way. Small increases of arterial AAs produce a disproportionate increase of myocardium AA uptake [1].

Here, we have hypothesized that patients with chronic heart failure (CHF) may have reduced arterial AA levels, restricting the supply to and availability of heart AAs. Various mechanisms may potentially be operating during CHF to impair arterial AAs, including inadequate protein-energy intake [17], body AA overconsumption, particularly in hypermetabolic states [17], increased remodeling activity of the heart [18] and lung [19] and finally, the development of pathogenic gut flora [20]. Understanding arterial AA levels could be useful to understand whether heart anabolic activity and remaining heart capacity of energy production are being threatened by low AA s and furthermore may allow us to correct altered AAs through diet and/or supplementation of specific free AAs.

The goals of this study were therefore to measure arterial AA levels in CHF patients, to relate them to clinical disease severity and left ventricular (LV) function and track the potential risks for disease progression from low arterial/heart AAs. Although possible alterations of arterial AAs may impact the metabolism of organs and tissues relevant to heart failure, we only examined the potential effects of AAs on myocardial metabolism.

## 2. Methods

### 2.1. Study Population

We enrolled subjects diagnosed with chronic heart failure (CHF) and consecutively admitted to our Institute to undergo right cardiac catheterization for heart transplantation evaluation [21]. The measured duration of the disease was 1 to 4 years. As an inclusion criteria, the patients had to be clinically stable for at least one month [17] and have had stable body weight over the preceding 3 months. Patients with diabetes, insulin resistance (Homeostatic Model Assessment (HOMA) Index > 2.5), chronic obstructive pulmonary disease, chronic renal failure documented before CHF diagnosis, dysthyroidism, malignancy, clinical signs of intestinal malabsorption such as steatorrhea, diarrhea, and current corticosteroid treatment were all excluded, as these conditions are usually associated with perturbations of AA circulation/metabolism. Of 79 subjects enrolled, 41 patients (51.9%) (34 males) fulfilled all these criteria. Twelve patients were in New York Heart Association (NYHA) II functional class, 19 in III and 10 in IV.

The technical medical ethics committee of the Scientific Institute of Montescano approved the study design and informed written consent was obtained from each patient before the study (Protocol No. 68-2008).

### 2.2. Protocol Procedures

Within the first five days of their hospital stay, all patients underwent the following measures in addition to routine bio-humeral variables, echocardiographic variables, including left ventricular ejection fraction (LVEF %), exercise test (bicycle) (not in IV class patients) with determination of oxygen consumption.

#### 2.2.1. Cardiac Catheterization

As described elsewhere [21], patients underwent right cardiac catheterization with a SWAN-GANZ-catheter for thermos-dilution, introduced via the internal jugular vein (Seldinger technique), fifteen hours after overnight fasting. Cardiac output (L min^−1^) was considered for the scope of this study. We also measured the Cardiac Index (CI, L min^−1^ m^−2^ body surface area), Stroke Volume (SV, mL beat^−1^) and Stroke Volume Index (SVI, mL beat^−1^ m^−2^ body surface area).

#### 2.2.2. Pro-B-Type Natriuretic Peptide (NT-Pro-BNP)

Venous N-terminal pro-B-type natriuretic peptide (NT-pro-BNP) concentrations were calculated using enzymatic immune-fluorescence assay (results in pg mL^−1^).

#### 2.2.3. Arterial Amino Acid Concentrations

Blood samples were collected from the radial artery (1 mL after rest of at least 30 min. This was to determine AA concentrations, using high performance liquid chromatography (HPLC) (results in µmol L^−1^) as described elsewhere [22]. Although we measured plasma concentrations of 21 AAs, here we only looked at those AAs that changed significantly.

#### 2.2.4. Nutritional Intakes

As nutrition is a determining factor that influences arterial AA levels, we calculated patient daily nutritional intakes by analyzing their 3-day food diary, following an accepted methodology described elsewhere [23]. Nutritional analysis was computed using a computer program. Energy (Kcal_Intake_ = Kcal_I_) and macronutrient intake (carbohydrates; proteins; lipids; all expressed in grams, g) were also related to the patients’ body weight (kg). Kcal_I_ was also expressed as a percentage of the patients’ resting energy expenditure (REE). Daily normal energy and protein intakes were set at ≥25 Kcal/kg and respectively ≥1.0 g/kg.

#### 2.2.5. Resting Energy Expenditure (REE)

The patient’s REE (Kcal/day and Kcal/kg body weight) was measured by determining oxygen consumption (VO_2_, mL/min) and carbon dioxide (VCO_2_, mL/min) production according to the following equation [24]:

REE (Kcal/day) = (3.9 × (VO_2_) + 1.1(VCO_2_)) × 1.44

REE value between 90–110% of the predicted value by the Harris-Benedict equation (H-B) indicated a normo-metabolic state.

REE/H-B > 110% denoted hyper-metabolism, REE/H-B < 90% indicated hypo-metabolism.

#### 2.2.6. Estimation of Calorie-Protein Adequacy

Calorie intake was considered adequate for body requirements when Kcal_I_ ≥ TEE, where Kcal_I_ = daily ingested calories; TEE (total energy expenditure) = REE × 1.3 and 1.3 = factor for daily physical activity [25]. Protein intake was considered adequate when ≥1.1 g/kg/day. 

#### 2.2.7. Double Product

Double product (DP) was calculated as systolic blood pressure (mmHg) times the heart rate (HR, beat min^−1^) [26].

#### 2.2.8. Anthropometrics

Body mass index (BMI, kg m^−2^) was calculated as body weight (kg) divided by height (in m^2^). Eight healthy subjects (6 males, 51 ± 9 years; BMI 27.1 ± 2.2 kg m^−2^) of the healthcare staff served as controls and underwent arterial AA concentrations, nutritional intake analysis and REE measurements. 

### 2.3. Statistical Analysis

The results are shown as the mean value ± standard deviation (SD). A Chi square test was used for dichotomous variables. Inter-group differences in anthropometrics, plasma AAs, nutritional intakes, hemodynamic variables were compared using analysis of variance (ANOVA) test and Fisher’s protected least significant difference (PLSD) test. The linear (Pearson *r* test) or curvilinear (Spearman *r* test) regression analysis was used to show any possible correlations between plasma AAs and hemodynamic, LVEF %, DP, NT-pro-BNP, body resting VO_2_ and REE. We set statistical significance at *p* < 0.05.

## 3. Results

The study showed that three NYHA class groups (Table 1) were similar for all measured variables, excluding class IV subjects whom, when compared with II-III classes, had lower BMI (*p* < 0.05), SVI (*p* < 0.05), CI (*p* < 0.05), LVEF % (*p* < 0.05).

### 3.1. Arterial Amino Acids

Table 2 describes the arterial AA concentrations in healthy controls and CHF patients, as an entire group, and after stratification into NYHA classes. Compared with controls, as a global population, CHF subjects had lower levels of aspartic acid (*p* < 0.001), glutamic acid (*p* < 0.01), cysteine (*p* < 0.001), methionine (*p* < 0.001), taurine (*p* < 0.001). Patients in II NYHA class had significantly reduced aspartic acid (*p* < 0.001), methionine (*p* < 0.001) and taurine (*p* < 0.05). These three AAs were also reduced in class III subjects, who also had lower levels of glutamic acid (*p* < 0.01) and cysteine (*p* < 0.001). These statistical differences did not change when the II and III classes were grouped together.

There were stark differences for class IV patients compared to both controls and class II; individual AA, total AAs (AAtot), essential AAs (EAAs), branched chain AAs (BCAAs), were all significantly lower. Compared to NYHA III class, IV class had lower arterial concentrations of AAtot, EAAs, BCAAs and 85.7% of the AAs individually considered.

### 3.2. Energy Expenditure and Adequate Nutrition

In Table 3, REE, nutritional intakes and estimated calorie intake adequacy (Kcal_I_ ≥ TEE) were reported both for healthy controls and the CHF population. The results showed that similar to healthy subjects, the patients as an entire population as well as class II-III patients were normo-metabolic. On the contrary, patients in class IV were found to be in a hypermetabolic state. Compared to healthy controls, the CHF population and class II-III patients had adequate calorie intakes, while for IV class subjects, ingested calories, while normal in absolute terms (29.2 ± 4.6 Kcal/kg), were inadequate for body needs. Protein intakes were adequate for healthy control and all CHF NYHA classes.

### 3.3. Relation between Arterial Amino Acid Levels and Left Ventricular (LV) Function

**Figure 1:**
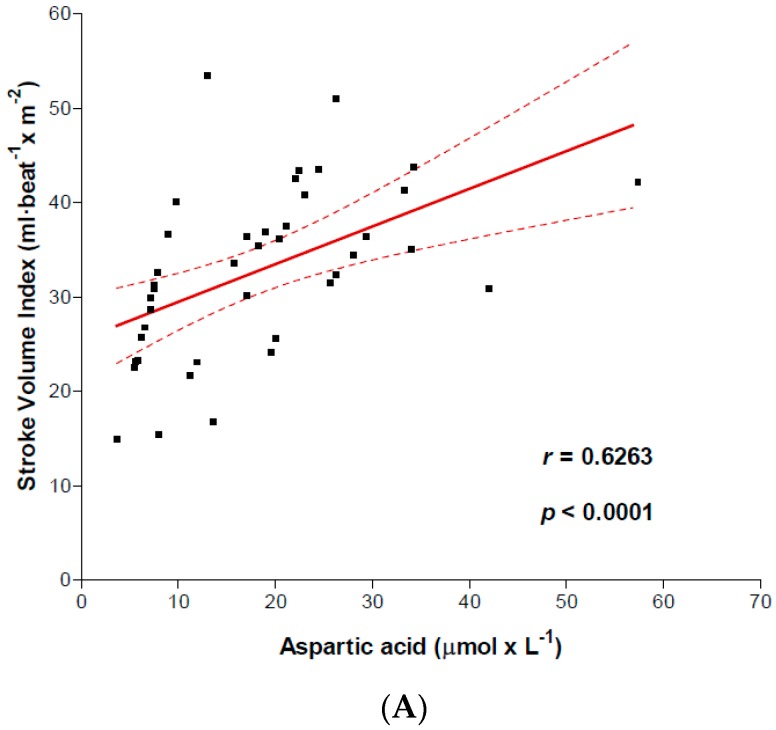

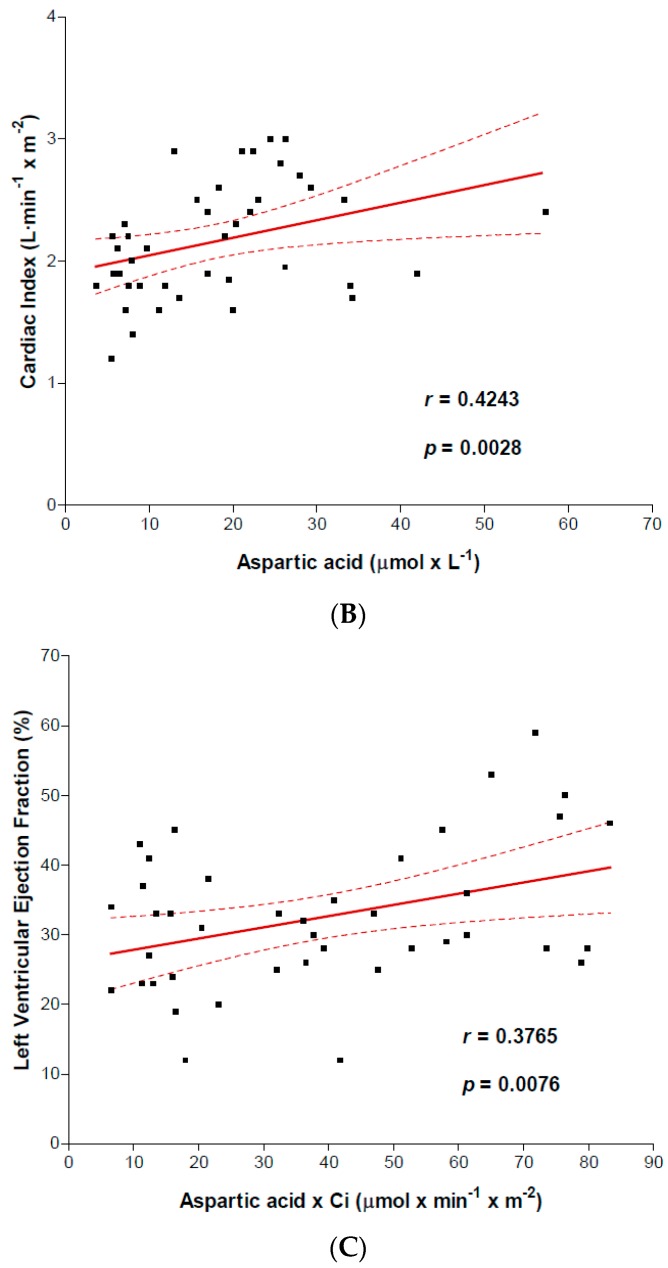
Relationships between arterial aspartic acid and left ventricular (LV) function. Correlation between Aspartic acid and stroke volume index (**panel A**), Cardiac Index (**panel B**). The (**panel C**) shows the correlation between arterial Aspartic acid times cardiac index and left ventricular ejection fraction.

**Figure 2:**
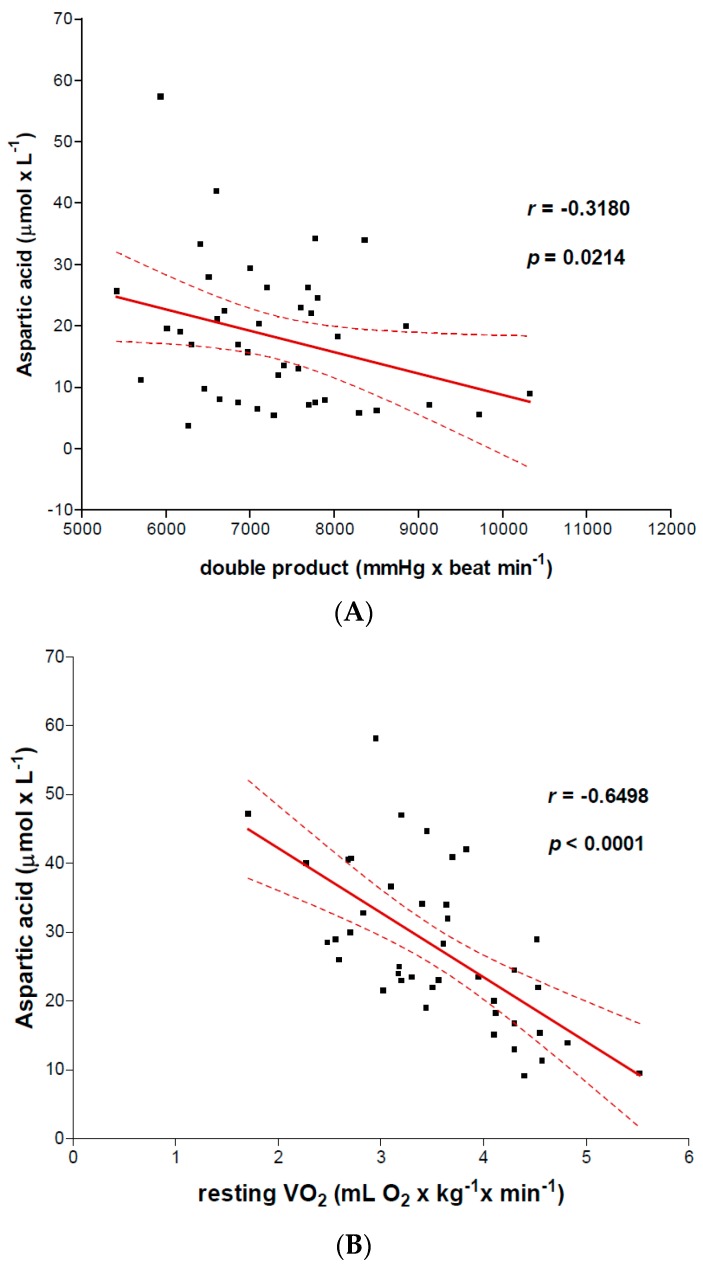
Negative correlation between arterial Aspartic acid levels and hemodynamic parameters. Arterial Aspartic acid negatively correlates with Double Product (**panel A**) and body resting VO_2_ (**panel B**).

We focused particularly on aspartic acid for correlations between AA levels and LV function. This AA plays a crucial role in cell oxidative metabolism and hence energy formation. The results showed several correlations (Figure 1 panels A, B, C) between arterial aspartic acid and systolic function (SVI, Spearman *r* = 0.6263; *p* < 0.0001), cardiac index (CI, Spearman *r* = 0.4243; *p* = 0.0028). Aspartic acid, expressed as aspartic acid concentrations per CI (µmol min^−1^ m^−2^), correlated positively with LVEF (%) (Pearson *r* = 0.3765; *p* = 0.0076). On the contrary, aspartic acid negatively correlated with DP (Spearman *r* = −0.3180; *p* = 0.0214) and body resting VO_2_ (Pearson *r* = −0.6498; *p* < 0.0001) (Figure 2 panels A and B). No significant correlations were noted between the other AAs and VO_2_. Moreover, the artery aspartic acid levels were associated with REE (Pearson *r* = 0.409; *p* = 0.046). 

Several AAs, expressed as the amount circulating each minute (AA × CI, µmol min^−1^ m^−2^), positively correlated with LVEF (%) (Table 4). 

Plasma NT-pro-BNP levels were negatively related to arterial AAtot, EAAs and namely to BCAAs/Leucine.

## 4. Discussion

This study shows that CHF patients may have reduced arterial AA levels, of which both their number and reduced rates are related to HF severity. Three AAs were found to be reduced in class II (aspartic acid, methionine, taurine), five in class III (aspartic acid, glutamic acid, methionine, cystein, taurine) and all AAs were reduced in class IV. Aspartic acid, methionine, taurine appear to delineate a characteristic profile not only for being low in each CHF class, but also for their progressive reduction with increased disease severity.

Furthermore, our results indicate that progressive reductions in arterial AA levels were associated with progressive LV deterioration. In class IV patients, low AA levels were also associated with hyper-metabolism and inadequate calorie intakes.

### 4.1. Mechanisms Underlying Low Arterial AAs

Multiple individual or variably combined mechanisms underlie these reduced arterial AA levels, including inadequate protein-calorie intake, the development of pathogenic gut flora and/or body/heart AA over-consumption. In addition, inter-individual variability of intestinal protein absorption may condition the impact of these mechanisms on arterial AA levels. Indeed, normal protein absorption is not complete (100%) but is usually between 66–95% [27].

In NYHA classes II-III, protein calorie ingestion was not responsible for impaired AA levels as it was adequate for the patients’ metabolic needs. On the contrary, in hypermetabolic class IV patients, calorie intake [17], which included calories derived from protein, while normal in absolute terms, was inadequate for the patients’ calorie requirements, possibly contributing to lower AA levels. We calculated (data not shown) that if the class IV patients had had adequate calorie intake, they would have ingested 14.4 g protein/day (0.20 g/kg) more. The negative calorie/protein balance in these patients was due to bodily excess of energy expenditure. This hyper-metabolism indicated that bisoprolol may reduce but does not totally counteract the excess of REE induced by HF-primed inflammation [28], dyspnea, breathing work and possible lipopolysaccharide translocation into the circulation [29,30] following gut permeability [20]. That bisoprolol was probably effective in reducing REE may be inferred by the fact that the entire CHF population, compared with subjects without β blockers [17], had 11.8% lower REE. 

We believe that the possible reduction of REE induced by bisoprolol could explain the weight gain found in CHF patients of the CIBIS II trial [31]. In class IV patients, weight stability, notwithstanding the negative calorie balance (−325 Kcal/day), probably occurred at the expense of daily physical activity [17].

The timing of protein-calorie inadequacy however, is crucial to impair arterial AA levels and is particularly important during acute patient decompensation, when adequate protein calorie ingestion is precluded for days or even weeks. In our clinical practice, indeed we calculated that patients, during hemodynamic instabilization, could ingest less than 500 Kcal/day and 18 g protein/day, very low nutrition to meet metabolic needs and limit the acute hyper-catabolic state. To prolong this hyper-catabolism, improved body protein metabolism after acute metabolic stress requires months of both compensatory nutritional intakes and clinical stability. These two aspects outline the dramatic impact of periods of heart acute decompensation that impair circulating AAs.

The development of pathogenic gut flora [20], occurring in more than three quarters of class II to IV patients, may contribute to lower plasma AAs. Indeed, pathogenic bacteria decrease the retrieval of calories of both non-digested foods and non-absorbed proteins [27].

Body and heart over-consumption may be a major mechanism contributing to reduce arterial AAs even during patient hemodynamic stability. In classes II-III, AA over-consumption would selectively regard pro-energy aspartic acid, glutamic acid, methionine and the antioxidants cysteine, (methionine) and taurine. In class IV patients, AA over-consumption may be accentuated by a hypermetabolic state and inadequate calorie intake, explaining the dramatic drop of all circulating AAs. In CHF, increased lung [19] and heart [18] protein metabolism remodeling leads to AA over-consumption. Clinically important, lung AA over-consumption is higher in CHF subjects without bisoprolol [19]. Here, we should note that under normal conditions the lungs are as important as skeletal muscle in maintaining plasma amino acid homeostasis [32].

Over-consumption of aspartic acid may be inferred by the negative correlation of this AA with body resting VO_2_ and myocardial energy consumption (DP) as well as by positive association with REE. Selective over-consumption of pro-energy and antioxidant AAs in class II and III may probably account for the co-existence of normal EAAs (except for methionine) with low non-essential AAs. Normal EAAs in class II-III patients indicate that protein ingestion by these patients was also qualitatively adequate. With respect to methionine, diet could contribute in reducing arterial levels as this essential AA is contained in relatively limited amounts in food.

### 4.2. Risk of Low Arterial AAs on Myocardial Energetics 

A shortage of heart AAs may aggravate heart energy deficit by altering certain important mechanisms underlying myocardial energy formation, such as mitochondrial TCA cycle [5,33,34] for FAOX and GLUOX, mitochondrial energy formation under anaerobic conditions [7,8,35], glycolytic activity [7] Correlations between arterial aspartic acid and other AAs with LV systolic function could suggest a link between AAs-associated LV energy content, contractility force and LV function. These correlations may explain the parallelism between reduced arterial AAs and progressive LV dysfunction.

Low methionine may further aggravate mitochondrial aerobic energy production. This is because this AA forms both carnitine, essential for FAOX [36], and creatine, essential for energy storage and transport to contractile myofibrils. In HF, myocardial creatine is reduced by as much as 60% [37]. 

### 4.3. Risk of Low Arterial AAs on Heart Oxidative Stress

Low cysteine, methionine, taurine may increase heart oxidative stress [38]. Low cysteine may be particularly damaging for the heart’s anti-oxidative capacity, not only because this AA is the precursor of glutathione, but also because the glutathione genes are upregulated in HF [12], to counter the oxidative stress associated with glucose-associated chronic mTOR activation and cardiac hypertrophy [12]. The finding of low cysteine in our study is in contrast with higher plasma cysteine levels observed in a recent investigation [39]. Method differences could explain this discrepancy, such as important age differences between controls and the heart failure patients and the presence of a large population with chronic renal disease described in this study.

Taurine is synthetized by methionine and cysteine. Therefore, in CHF, low taurine may be caused by impairment of these precursors as well as inflammation and oxidative stress [40,41]. Taurine has anti-oxidant properties, so low taurine may increase patient oxidative stress as well as causing intracellular and mitochondrial calcium overload [42]. The positive effects of taurine supplementation on symptoms and LV function of CHF patients have been amply documented in a number of studies [42,43,44,45].

### 4.4. Risk of Low Arterial AAs on Heart Protein Metabolism Remodelling

In HF, the ventricle wall is in continuous anabolic activity [3,10,12] inducing high AA/EAA consumption. Among EAAs, BCAAs and in particular leucine [46] have all been found to be strong anabolic agents [3]. As a result, the negative relation between serum pro-NT-BNP levels, markers of hypertrophied heart and arterial BCAA/leucine [21] would suggest a relation between intense heart remodeling, BCAA overconsumption, and impoverishment in arterial BCAAs. Thus, low heart availability of EAAs/BCAAs does not meet ventricle anabolic requirements and leads to heart catabolic activity prevalent over anabolic activity (hyper-catabolism). Of practical importance, even in healthy hearts overnight fasting induces a negative protein balance with a 4–5% loss of myocardial protein mass [3].

Under fasting conditions, normal arterial BCAA levels are fundamental to limit overnight protein degradation and induce protein synthesis [3]. This fact highlights the great importance of normal arterial BCAAs for the myocardial structure. In HF, hyper-catabolism may be devastating as net protein degradation causes ventricular wall thinning, reduces the ventricle resistance to elevated pressure development and mechanical stress and accentuates myocardial contractile dysfunction [47]. It has been shown that plasma alteration in AAs, in particular BCAAs, can mediate the effects of protein-energy malnutrition on cardiac functions, for example in cardiac cachexia. Of note, impaired LV function in malnourished children may be partially reversed by nutrition rehabilitation [48].

Our results suggest that class II-III patient hearts are protected from maladaptive protein remodeling, whereas this was not the case for class IV patients. Alterations in heart hemodynamics are not without negative effects on arterial AA levels (creating a vicious cycle). Indeed, CHF-induced alterations in bowel perfusion and edema can lead to protein loss [49]. 

This study confirms our previous results [21], which documented a reduction in EAAs in non-obese CHF subjects. At the same time, this study provides additional information as it shows the disease severity-related decline of arterial levels of those non-essential (and essential methionine) AAs with the greatest impact on myocardium energetics, anti-oxidative capacity and myocardial protein remodeling.

**Figure 3:**
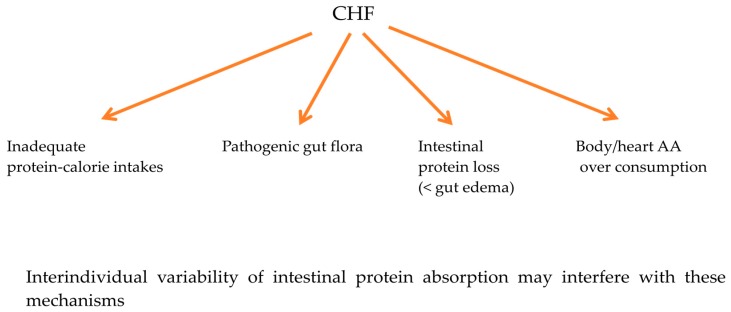
Synthesis of mechanisms for reduced arterial amino acids levels in clinically stable chronic heart failure (CHF).

Table 5 and Table 6 and Figure 3 summarize the mechanisms underlying reduced AA alterations in the three NYHA classes, and potential risks for myocardium metabolism.

## 5. Limitations of This Study

The NYHA classification itself is a limitation given that it is a subjective measure of heart failure but it is of routine clinical importance especially in subjects with exercise intolerance that precludes the achievement of VO_2_ max. This study did not investigate myocardial use of amino acids. This issue requires an appropriate targeted study on myocardial net AA balance, for example by measuring arterial-coronary sinus differences. Understanding patient body composition would have strengthened the discussion, even though the study clearly shows a trend towards reduced BMI linked to the worsening of clinical-functional classes. Plasma levels of energy substrates affecting heart metabolism such as lactate, pyruvate, and ketone bodies were not measured in this study.

## 6. Future Directions for Clinical Practice

Before correcting, in clinical practice, reduced plasma amino acids with specific amino acids for specific NYHA class, there is a need to investigate these results in a large CHF population. This would need to address the myocardial use of amino acid in CHF subjects both with and without supplemented amino acids. All these studies are fundamental to avoid excess circulating amino acids that could be a risk for cardiovascular disease events.

An important, recent review [50] reports the association of high plasma levels of the amino acid phenylalanine, glutamate, BCAA with CVD (Cardiovascular Disease) risks as well as with cardiovascular risk factors including obesity, insulin resistance and diabetes mellitus. These associations seem to rely on complex mechanisms such as inflammation and oxidative stress. These amino acids did not increase in the study patients but serve to underline the need to avoid inappropriate excessive amino acid administration.

In the meantime, however, our results highlight the need for a strict nutritional survey and dietetic education of CHF patients, supplementary to standard dietary advice for these subjects.

1. The intake of protein with high biological value providing adequate amounts of EAAs should be ensured in CHF patients and mandatory in NYHA class IV patients. If these proteins are not adequately ingested, a supplementation with physiological doses of EAAs (7–8 g/die) can compensate for deficient intake of an individual serving of protein with high biological value; for example 7–8 g of free essential amino acids are the equivalent to the amount of EAAs contained in 500 mL of milk.
  Protein ingestion should be supplemented with free essential amino acids in subjects in IV NYHA class association, particularly during the metabolic recovery after acute decompensation. Normal protein intake requires too much time to restore reduced circulating amino acids.
2. Given the mutual influence between inflammation and oxidative stress, patient selection of food with anti-inflammatory and antioxidant properties could be useful for CHF patients from II to IV NYHA classes.
  Oatmeal, milk, orange juice and raisins may all prevent postprandial endotoxemia [51] as well as a rise in various markers of oxidative stress. Orange juice neutralizes [52] the pro-inflammatory effect of high fat, high carbohydrate intakes, typical of western diet. High amounts of cystin and methionine are contained in wheat, toasted peanuts, red meat, rabbit, sardines, egg and chicken. Fruits, vegetables, tea and wine contain flavonoid antioxidants (quercetin, myricetin, apicetin and luteolin). The intake of the potent antioxidant vitamins (A, C and E) as well as enzymin factors (zinc, selenium and copper), should be recommended in all CHF subjects.

## Figures and Tables

**Table 1:** Demographic, anthropometric and clinical characteristics, functional class, etiology, biohumeral variables, physical performance (bicycle test), and cardiac hemodynamic variables of the studied CHF patients.

		All CHF (*n* = 41)	NYHA II (*n* = 12)	NYHA III (*n* = 19)	NYHA IV (*n* = 10)
Demographic	Age (years)	60 ± 8.2	57 ± 13.1	57.0 ± 8.4	61 ± 3.9
	Sex (male/female)	30/11	8/4	12/7	8/2
Anthropometrics	Body weight (kg)	78.5 ± 15.2	82 ± 21.3	82.1 ± 14.7	73 ± 2.8
	BMI (kg m^−2^)	27.7 ± 5	29.2 ± 5.5	28.8 ± 5.5	24 ± 0.7 *
Etiology	Ischemic	21 pts (51.2%)	6/12	8/19	7/10
	Idiopathic dilated cardiomyopathy	13 pts (31.7%)	4/12	6/19	3/10
	Valvular	7 pts (17.1%)	2/12	5/19	0/10
Functional class	NYHA	2.9 ± 0.7	2 ± 0	3 ± 0	4 ± 0
SBP/DPB	Blood pressure	109.7 ± 14.7	117 ± 16.9	107 ± 13	105 ± 14.3
Systolic blood pressure mmHg/diastolic blood pressure mmHg		67 ± 14.5	71 ± 13.5	68 ± 14	63 ± 16
Medications	ACE inhibition	41 pts (100%)	12 pts	19 pts	10 pts
	β blockers	41 pts (100%)	12 pts	19 pts	10 pts
	Digoxin	13 pts (31.7%)	5 pts	3 pts	5 pts
	Diuretics	41 pts (100%)	12 pts	19 pts	10 pts
	Spironolactone	4 pts (9.7%)	-	1 pts	3 pts
Blood	Glucose (mg dL^−1^; nv = 80–110)	97.5 ± 18.1	92 ± 15.5	102.5 ± 20.9	98.5 ± 19.1
	Albumin (g dL^−1^; nv = 3.5–5)	4.4 ± 0.4	4.5 ± 0.4	4.5 ± 0.5	4.4 ± 0.1
	Hemoglobin (g dL^−1^; nv = 12–15)	13.0 ± 12.0	12.4 ± 1.7	12.7 ± 1.6	11.3 ± 3.2
	Sodium (mEq L^−1^; nv = 135–145)	135.4 ± 5.1	138.4 ± 2.9	135 ± 5	137 ± 2.8
	Potassium (mEq L^−1^; nv = 3.5–5.0	4.0 ± 0.5	4.3 ± 0.2	3.9 ± 0.6	3.7 ± 0.1
	Creatinine (mg dL^−1^; nv = 0.6–1.2)	1.5 ± 0.5	1.3 ± 0.4	1.6 ± 0.7	1.3 ± 0.1
	Urea (mg dL^−1^; NV = 20–40)	78.8 ± 58.2	67 ± 38.4	88.4 ± 75.6	62.5 ± 30.4
	NT-pro-BNP (pg mL^−1^; nv < 125 for age < 75 years)	1680.6 ± 983.6	347 ± 215.6	2022.3 ± 813.5	2699 ± 1750
Physical performance	VO_2_ rest (mL O_2_ kg^−1^ min^−1^)	3.4 ± 1	3.2 ± 0.01	3.4 ± 1.1	4.1 ± 1.4
	VO_2_ peak (mL O_2_ kg^−1^ min^−1^)	12.4 ± 3	12.1 ± 1.0	11.2 ± 2.3	nd
	HR peak (beat min^−1^)	109.2 ± 22.7	111.3 ± 11.7	95.2 ± 22.1	nd
Hemodynamic variables	CO (L min^−1^)	3.9 ± 1.1	4.7 ± 1.4	4 ± 1.0	3.0 ± 1.6
	CI (L min^−1^ m^−2^)	2.2 ± 0.5	2.4 ± 0.4	2.2 ± 0.5	1.9 ± 0.3 £
	SV (mL beat^−1^)	57.5 ± 19.5	66 ± 26.8	61.6 ± 19.3	49.5 ± 30.4
	SVI (mL beat^−1^ m^−2^)	32.1 ± 8.9	34.3 ± 7.4	34.2 ± 9.7	25.5 ± 5.5 £
	LVEF (%; nv > 55)	32.4 ± 10.4	39.3 ± 10.7	33.5 ± 8.3	27.5 ± 8.9 *£
	WP (mmHg)	19.2 ± 9.3	15.4 ± 7.6	17.6 ± 9.5	24.8 ± 11

Abbreviations: CHF = chronic heart failure; BMI = body mass index; pts = patients; CI = cardiac index; CO = cardiac output; HR = heart rate; LVEF = left ventricular ejection fraction; NT-pro-BNP = N-terminal pro-B-type; natriuretic peptide; nd = not determined; nv = normal value; NYHA = New York Heart Association; SV = stroke volume; SVI = stroke volume index; VO_2_ = oxygen consumption. WP = Wedge Pressure; Data are given as mean ± SD. Statistical analysis: ANOVA test and Fisher’s PLSD test; Statistical significance was set at *p* < 0.05. IV NYHA class vs. II class * *p* < 0.05; III class £ *p* < 0.05.

**Table 2:** Arterial concentrations of amino acids (µmol L^−1^) in healthy subjects and in CHF patients before and after NYHA categorization.

	HEALTHY	CHF			
		All-CHF	NYHA		
	(*n* = 8)	(*n* = 41)	II (*n* = 12)	III (*n* = 19)	IV (*n* = 10)
AAtot	2530.7 ± 55.84	1954 ± 1042	2459 ± 108	2318 ± 1075	655.6 ± 97.34 ‡•&
EAAs	616.2 ± 20.3	601 ± 356.01	727.5 ± 110	748.4 ± 370.1	169.4 ± 26.59 †•&
BCAAs	286.5 ± 13.57	264.9 ± 160.7	310.6 ± 62.89	331.5 ± 174.8	83.73 ± 15.49 *∞&
Aspartic acid	112.1 ± 8.858	16.41 ± 11.44 ‡	23.47 ± 5.527 ‡	17.16 ± 13.51 ‡	6.511 ± 1.492 ‡•
Glutamic acid	198.6 ± 10.61	134.0 ± 54.22 †	191.5 ± 20.46	126.7 ± 47.52 †	78.67 ± 11.22 ‡•&
Asparagine	61.04 ± 1.987	49.29 ± 21.86	60.18 ± 2.512	55.56 ± 23.75	24.32 ± 7.299 ‡•
Serine	88.39 ± 4.251	84.38 ± 52.34	93.51 ± 8.853	111.1 ± 55.73	22.71 ± 5.196 *∞£
Glutamine	464.9 ± 13.98	397.8 ± 267.9	498.8 ± 55.49	498.1 ± 292.8	86.15 ± 32.68 †•£
Hystidine	58 ± 5.155	50.78 ± 34.9	62.92 ± 8.836	65.17 ± 36.72	8.868 ± 5.402 †•£
Glycine	268.3 ± 11.97	205.2 ± 118.3	265.8 ± 14.06	247.3 ± 116.8	52.33 ± 5.963 ‡•£
Threonine	111.6 ± 7.3	77.34 ± 47.37	114.7 ± 13.63	84.82 ± 43.79	18.27 ± 5.284 ‡•£
Citrulline	24.58 ± 3.661	28.83 ± 20.65	28.49 ± 6.965	39.66 ± 22.65	6.471 ± 1.171 ∞&
Alanine	312.6 ± 15.67	236.6 ± 145.5	324.9 ± 23.35	274.3,4 ± 149.9	59.08 ± 6.012 ‡•&
Arginine	59.28 ± 7.607	52.89 ± 23.35	61.53 ± 7.889	61.71 ± 24.54	25.75 ± 7.758 †•&
Tyrosine	56.25 ± 6.112	51.14 ± 29.92	57.29 ± 5.224	65.29 ± 32.81	16.87 ± 3.573 *∞&
Cystein	77.13 ± 5.139	36.57 ± 19.48 ‡	60.88 ± 8.241	31.69 ± 11.90 ‡	16.69 ± 6.602 ‡•
Valine	160.0 ± 15.8	145.5 ± 87.75	173.6 ± 38.04	178.1 ± 97.26	49.83 ± 8.432 ∞&
Methionine	9.7 ± 2.8	4.872 ± 1.861 ‡	7.196 ± 1.18 ‡	4.244 ± 1.086 ‡	3.279 ± 0.692 ‡•
Tryptophan	50.1 ± 4.9	39.93 ± 23.77	50 ± 5.481	49.10 ± 24.36	10.43 ± 3.011 ‡•&
Phenylalanine	51.3 ± 5.1	44.67 ± 27.75	50.25 ± 7.518	58.51 ± 29.09	11.68 ± 2.327 *∞&
Isoleucine	47.4 ± 4.1	41.83 ± 26.52	48.33 ± 6.597	53.79 ± 28.88	11.28 ± 2.949 *∞&
Leucine	79.1 ± 8.5	74.72 ± 48.84	90.21 ± 20.51	92.37 ± 53.13	22.62 ± 4.539 ∞&
Lysine	107 ± 114	119.9 ± 72.45	137.6 ± 33.55	154.0 ± 73.57	33.8 ± 5.485 ∞&
Taurine	133.3 ± 23.03	86.02 ± 28.26 ‡	97.17 ± 14.14 *	93.86 ± 29.25 †	57.75 ± 20.26 ‡∞@

Abbreviations: CHF = chronic heart failure; NYHA = New York Heart Association categories; AAtot = total amino acids; BCAAs: branched chain amino acids (leucine, isoleucine, valine); EAAs = essential amino acids (BCAAs+ threonine, methionine, tryptophan, phenylalanine and lysine). Data are given as mean ± SD. Statistical analysis: ANOVA test and Fisher’s PLSD test. Statistical significance was set up at *p* < 0.05. Patients vs. Healthy: * *p* < 0.05; † *p* < 0.01; ‡ *p* < 0.001. IV NYHA class vs. II class § *p* < 0.05; ∞ *p* < 0.01; • *p* < 0.001. III class £ *p* < 0.05; @ *p* < 0.01; & *p* < 0.001.

**Table 3:** Predicted energy expenditure (H-B), resting energy expenditure (REE) and nutritional intakes in chronic heart failure (CHF) patients and in healthy controls.

		HEALTHY	CHF			
			All CHF	NYHA		
				II	III	IV
Weight		66.4 ± 8.6	78.5 ± 15.2	82.2 ± 21.3	82.11 ± 14.7	73.4 ± 2.8
H-B	Kcal/day	1351 ± 120	1568 ± 185 *	1561.5 ± 245 *	1534 ± 251 *	1677.7 ± 209 *
REE:						
	Kcal/day	1384 ± 127.8	1584 ± 195	1637.7 ± 273	1532.1 ± 202	1890.7 ± 151
	REE/H-B (%)	102.4 ± 0.8	101 ± 5.7	104.9 ± 3.8	99.8 ± 3.3 **	113 ± 1.3 **
	Kcal/kg	20.2 ± 0.7	20.2 ± 3.7	20.0 ± 3.9	18.7 ± 4.9	25.9 ± 3.1 *
Nutritional Intakes:						
	Energy:					
	Kcal I/day	1797 ± 195	2220 ± 393.3	2361.6 ± 361	2167.4 ± 337	2132 ± 482
	Kcal I/kg	28.5 ± 1.1	28.1 ± 5.0	28.8 ± 4.4	26.4 ± 4.1	29.2 ± 4.6
	CHO:					
	g/day	264.6 ± 31	271.8 ± 11.4	280.6 ± 17.8	275.9 ± 13.3	259.8 ± 3.1
	g/kg	4.2 ± 0.5	3.3 ± 0.9	3.4 ± 0.8	3.4 ± 0.9	3.6 ± 1.1
						
	Proteins:					
	g/day	70 ± 12	92.9 ± 10.5	92.7 ± 13.1	92 ± 11.5	94.35 ± 7.1
	g/kg	1.1 ± 0.2	1.18 ± 0.2	1.1 ± 0.2	1.12 ± 0.14	1.31 ± 0.31
	Lipids:					
	g/day	51 ± 12	84.0 ± 10.6	95.9 ± 5.3	77.1 ± 9.9	79.3 ± 16.6
	g/kg	0.8 ± 0.2	1.0 ± 0.2	1.2 ± 0.2 *	0.9 ± 0.1	1.1 ± 0.2
Adequacy of calorie Intakes						
	Kcal I/TEE (%)	130 ± 1.2	140.1 ± 3.8 **	144.2 ± 5.2 **	141.4 ± 4.3 **	112.8 ± 4.1 ∞ **

H-B = Harris-Benedict equation; REE = Resting energy expenditure; TEE (Total energy expenditure) = REE × 1.3. Data are given as mean ± SD. Statistical analysis: ANOVA test and Fisher’s PLSD test. Statistical significance was set at *p* < 0.05 vs. Healthy controls: * *p* < 0.05; ** *p* < 0.01 vs. II-III NYHA groups ∞ *p* < 0.01.

**Table 4:** Significant correlations between arterial amino acids × CI (µmol min^−1^ m^−2^) and left Ventricular Ejection Fraction (%).

Amino Acids	*p* Value
Asparagine	0.052
Serine	0.006
Glutamine	0.02
Threonine	0.02
Alanine	0.0499
Arginine	0.127
Tyrosine	0.0139
Valine	0.0227
Tryptophan	0.0134
Phenylalanine	0.0184
Leucine	0.0086
Lysine	0.018
AA tot	0.0128
EAA tot	0.014
BCAA	0.0185

Correlation test: Spearman *r* test. Statistical significance at *p* < 0.05. CI = Cardiac Index. AA tot = Total Amino Acids. EAA = Essential Amino Acids. BCAA = Branched Chain Amino Acids.

**Table 5:** Reduced plasma amino acids (AAs) in clinically stable chronic heart failure (CHF).

Severity (NYHA)	Reduced Amino Acids
II class	Aspartic acid methionine, taurine
III class	Aspartic acid, methionine, taurine. Glutamic acid, cysteine
IV class	All the standard amino acids

NYHA = New York Heart Association categories

**Table 6:** Potential risks for myocardium metabolism from reduced arterial amino acids (AAs) in chronic heart failure (CHF).

Severity (NYHA)	Effects on Myocardium Metabolism
II and III classes	Aggravation of mitochondrial energy production Increased heart oxidative stress
IV class	The above + alterations in protein metabolism remodelling
The biochemical effects may potentially impact on heart contractility, function	

NYHA = New York Heart Association categories.
